# Assessment Synergistic Effects of Integrated Therapy with Epigallocatechin-3-Gallate (EGCG) & Arsenic Trioxide and Irradiation on Breast Cancer Cell Line

**DOI:** 10.18502/ijph.v49i8.3901

**Published:** 2020-08

**Authors:** Vahid CHANGIZI, Samayeh AZARIASL, Elahe MOTEVASELI, Saeedeh JAFARI NODOOSHAN

**Affiliations:** 1. Department of Technology of Radiology and Radiotherapy, School of Allied Medical Sciences, Tehran University of Medical Sciences, Tehran, Iran; 2. Department of Molecular Medicine, School of Advanced Technologies in Medicine, Tehran University of Medical Sciences, Tehran, Iran; 3. School of Advanced Technologies in Medicine, Tehran University of Medical Sciences, Tehran, Iran

**Keywords:** Epigallocatechin-3-gallate, Arsenic trioxide, Apoptosis

## Abstract

**Background:**

Breast cancer is the most common invasive malignancy among women in the world. The current breast cancer therapies pose significant clinical challenges. Low-dose chemotherapy represents a new strategy to treat solid tumors in combination with natural products such as green tea catechins. Epigallocatechin-3-gallate (EGCG) is the major polyphenolic extract from green tea with potent anticancer and antioxidant effects. The purpose of this study was to investigate the effects of EGCG, Arsenic trioxide (ATO) and gamma radiation on MCF-7 cell line.

**Methods:**

The anti-proliferative effects of EGCG and ATO individually, moreover in combination with radiation on MCF-7 cells were evaluated with MTT assay. The expression of apoptotic gens (Bax, Bcl-2, Caspase-3 and Fas) was assessed by real-time PCR.

**Results:**

Based on the results of MTT assay, EGCG and ATO exhibited dose and time-dependent anti-proliferative effects on MCF-7 cells. The combined therapy of EGCG and ATO in presence and absence radiation could rise cell death up to 80%. Moreover, integrated therapy made Bax up-regulated and Bcl-2 down- regulated.

**Conclusion:**

In assessment synergistic effects of integrated therapy with EGCG and ATO and irradiation had been significant impact on low dose chemotherapy for breast cancer treatment.

## Introduction

Breast cancer is the most common form of cancer among women and also causes the secondhighest morbidity rate worldwide (1, 2). The incidence rate of breast cancer is rising annually in the world (3). Unfortunately, the average age of breast cancer was decreased to less than 40 yr old in Iranian women in recent years (4, 5).

The treatment of malignant and cancerous tumors is considered as one of the crucial purposes of medical society so that various treatment protocols have been conducted. Radiotherapy, chemotherapy and surgery are the common treatment methods. Mostly surgery could not remove the entire tumor. Radiotherapy and chemotherapy could damage surrounding normal tissues (6). To reduce harmful effects of radiation and chemotherapy, this study aimed to investigate the integrated therapy by combination of therapeutic properties of plants, radiotherapy and chemotherapy on MCF-7 breast cancer cell line.

Tea is the most popular beverage in many countries, next to water. Green tea (Camellia Sinensis) contains at least four green tea catechins and among the polyphenols present in green tea (about 30% of the total weight of tea leaves), epi-gallocatechin-3-gallat (EGCG), is the most (7–11). Anti –tumorigenic properties attributed to EGCG include inhibition of cell proliferation and tumor growth, inhibition of tumor invasion and angiogenesis and cell cycle arrest. This type of catechin has been shown to inhibit cancer process in vitro and in vivo, in a high variety of cancer types including breast, skin, colorectal, pros tat, lung and liver cancer (12,13). Besides, anti-oxidant effects of EGCG can help us to improve the adverse side effects of cancer therapy. Therefore, EGCG can be combined with chemotherapy drugs to reduce the toxic effects of them (14, 15).

Arsenic trioxide (AS_2_O_3_) has been used since long times ago to treat a variety of diseases such as tooth marrow diseases, psoriasis and syphilis. The first report of anti-cancer associated with this drug was for leukemia in china in 1800 (16). Nowadays, Arsenic trioxide (ATO) is also used clinically to treat acute promyelocity leukemia. Moreover, it has been recently applied to treat solid tumors like breast cancer with induction of apoptosis (16, 17). Integrated therapy of ATO with other drugs, may provide a new chemotherapeutic solution for the treatment of breast cancer (17).

DNA is a critical target for ionizing radiation (IR) since it includes gens conducting cell proliferation and cell growth so that it’s with damage could reduce the cell viability severity. Therefore, the ionizing radiations (IR) have potential to induce DNA damage and apoptosis (18, 19).

Programmed cell death (PCD) pointing to apoptosis, autophagy and programmed necrosis, is to make statement death of a cell in any pathological format with an intracellular program. First-time apoptosis was introduced by Kerr to describe the physiological cell death in eukaryotes. Apoptosis is performed during embryonic stage and tumor suppression (20–22).

We aimed to investigate the effects of EGCG, Arsenic trioxide (ATO) and gamma radiation on MCF-7 cell line.

## Materials and Methods

### Cell Lines and Reagents

MCF-7 Cell Line, noninvasive estrogen receptor (ER) positive was prepared from the Pasteur Institute (Tehran, IRAN). Trypsin, EGCG, 3-(4, 5-dimethylthiazol-2-yl)-2, 5-diphenyl tetrazolium (MTT) were purchased from Sigma (Germany). EGCG dissolved in dimethyl sulfoxide (DMSO, Solon, OH, USA). Arsenic Trioxide (ATO) was purchased from Merck (Germany) and dissolved in Phosphate buffered saline (PBS) (Gibco, UK), at a concentration of 1mM at a stock solution and diluted before use.

### Cell Culture

Human breast cancer cell line MCF-7 cultured in the RPMI 1640 with 10% (V/V) heat-inactivated (at 56 °C for 30 min) fetal bovine serum (FBS), (100 units/mL penicillin and 100 mg/mL streptomycin at 37 °C under 5% CO_2_.The medium was changed every day, and cells were passed using trypsin/EDTA. Cells in the logarithmic growth period were selected for experimental studies.

### Drug and radiation treatment

In combination treatment, MCF-7 cells were treated by concentration of EGCG or ATO, then after 24 h, the cells were treated with γ-radiation at a dose of 2 Gy. About 24 h later, cells were used for analyses of the MTT assay or Real time PCR. Radiation was performed using cobalt 60 source with a source- to- plate distance of 80 cm and a dose rate of 100 cGy/min. Experiments were repeated three times.

### MTT assay

The MCF-7 cells (10000cells/well) were incubated in the 96-well plate for 24 hours. Different concentrations of 10, 30, 50, 70, 90 and 100 μM for the EGCG and 4, 5, 6, 7, 8, 9, 10 and 15 μM for the ATO were examined separately to get IC_50_ (inhibition concentration for MCF-7 at the minimum possible time). On this base, the incubation times for EGCG and ATO were used 24 h and 48 hours. Then the medium was being removed and cells were incubated with 20 μL (0.5 mg/mL dissolved in PBS) MTT and medium cultured 37 °C for 4 hours. Then culture medium containing MTT was removed and 100 μL dimethyl sulphoxide (DMSO) was added to solubilize the blue formazon by viable cells. Plates were read using an ELISA plate reader at 570 nm.

Cell viability is expressed as a percentage rate of exposed cells to control cells.

$$\begin{array}{l} \text{Survival}\;\text{rate}\;\text{of}\;\text{tumor}\;\text{cells}\left(\%\right)\;=\\ \frac{\mathrm{Expremental}\;\mathrm{groupA}\;\mathrm{value}}{\mathrm{Control}\;\mathrm{groupA}\;\mathrm{value}}\times 100 \end{array}$$

All steps mention above were repeated three times for EGCG and ATO separately. As resultant concentrations of 50 μM for EGCG and 7 μM for ATO were selected as the optimized concentrations. Then 6 groups of MCF-7 were selected for treatment as follows: The first group was selected as a control group not taken any treatment, second group was irradiated by 2 Gy Gamma radiation (cobalt 60 source), the third group was treated with concentrations of EGCG in the presence of 2 Gy Gamma. Then the forth group was treated with concentration of ATO in the presence of 2 Gy Gamma, and finally fifth and sixth groups were treated with combination of EGCG and ATO in the presence or absence of 2 Gy Gamma after 48 h.

### RNA extraction and cDNA synthesis

Total cellular RNA was obtained from MCF-7 cells. A commercial RNA isolation kit (Ribo Ex^TM^ LS, Gene All, China) was used to extract total RNA from 5 × 10^6^ cells of all groups (control, EGCG 50μM, ATO 7 μM, 2 Gy, EGCG 50 μM +2Gy, ATO7 μM +2Gy, EGCG 50 μM +ATO 4 μM and EGCG 50 μM +ATO 4 μM +2Gy) according to the manufacturer’s instructions. The quality and quantity of extracted RNA samples were assessed using the Nanodrop ND 1000 (Thermo Scientific, USA). Based on the quality and quantity of extracted RNA, 500 ng total RNA in a 20 μl reaction (25ng/μl) was synthesized into cDNA using AccuPower Cycle Script RT PreMix (Bioneer, Korea) according to the manufacturer’s instructions.

### Real-time PCR

For real-time PCR analysis, eight groups were processed. The quantification of the selected genes by real-time PCR was performed using Rotor-Gene Q (QIAGEN Hilden, Germany) using the primers shown in Table 1. Every reaction consisted of 1μl cDNA (1:20 dilution), 1 μl of each primer (F=0.5, R=0.5), 5μl reaction buffers (AMPLIQON Master Mix Green) and 3 μl H_2_O (total reaction volume 10μl) (Invitrogen). Real-time PCR cycles consisted of 2 min at 50 °C, 4 min at 95 °C for polymerase activation, 40 cycles of 15 sec at 94 °C (denaturation), 30 sec at 60 °C (annealing) and 30 sec at 72 °C (extension). Finally, melting was carried out at 55 °C–98 °C (1 °C in cerements) for 90 sec of pre-melt conditioning on first step and 5 sec for each step.

**Table 1:** Real-time PCR primers used in this study

***Gene***	***Primer***
Bax (F)	5CGCCGTGGACACAGACTC3
Bax (R)	5TCCCGGAGGAAGTCCAATGT3
Bcl-2(F)	5GACTTCTCCCGCCGCTAC3
Bcl-2(R)	5ATCTCCCGGTTGACGCTCT3
Fas (F)	5GACCCTTGCACCAAATGTGA3
Fas(R)	5AAGACAAAGCCACCCCAAGT3
Casp-3(F)	5TTGATGCGTGATGTTTCTAA3
Casp-3(R)	5AATGCCACAGTCCAGTT3

F: Forward

R: Reverse

### Data analysis

The threshold cycle CT) of each sample was normalized by using 2^−ΔΔCT^ method into house- keeping gene. Relative quantification analysis was carried out with the Rotor Gene Q series software version 2.3.1. The threshold cycle (CT) values provided by Real-time PCR were used to calculate the relative fold expression according to the 2^−ΔΔCT^ method (23) and REST 2009 software (version 2.0.13). All experiments were performed twice. Comparison among different groups was performed by using ANOVA test. A value of *P*≤0.05 was considered to be statistically significant.

## Results

Changes in survival Rates of MCF-7 Cells.

**Fig. 1:**
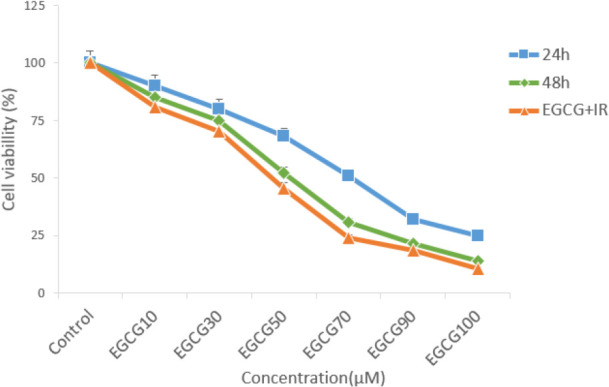
Survival rate of MCF-7 cells with different concentration of EGCG in 24 h, 48h and combinational with 2 Gy radiation measured by MTT

**Fig. 2:**
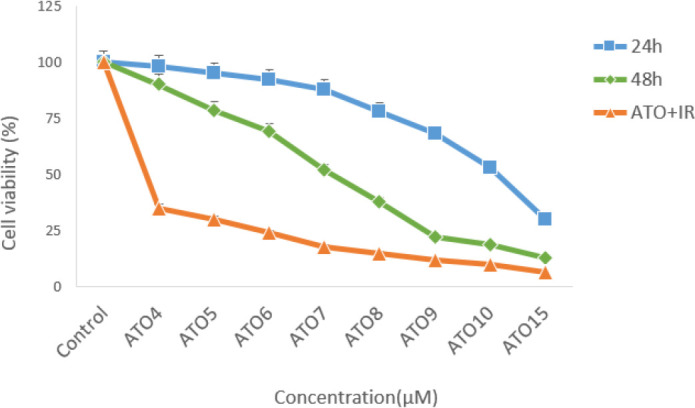
Survival rate of MCF-7 cells with different concentration of ATO in 24h, 48h and combinational with 2 Gy radiation measured by MTT

**Fig. 3:**
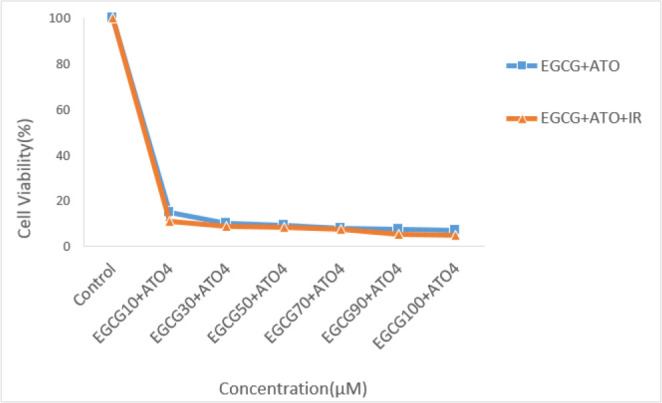
Survival rate of MCF-7 cells with different concentration of EGCG and 4 μM of ATO with and without 2 Gy radiation

MTT showed EGCG and ATO decreased cell viability of MCF-7 cell line with increase of dose and time. IC_50_ for this cell line was obtained for 70 μM EGCG after 24 h and 50 μM after 48 h treatment (*P*≤0.05) (Fig. 1). Moreover, the combined therapy of 2Gy gamma radiation with different concentration of EGCG was measured by MTT assay (*P*≤0.05) (Fig. 1). IC_50_ was measured for treatment with 7 μM ATO after 48 h (*P*≤0.05) (Fig. 2). The combined therapy of 2Gy gamma radiation with different concentration of ATO was measured by MTT assay (*P*≤0.05) (Fig. 2). The combined therapy of 4 μM ATO and different concentration of EGCG with and without 2 Gy gamma radiation after 48 h could cause cell death up to 80% for MCF-7 cell line (*P*≤0.05) (Fig. 3). Also that was greater than 33% cell death for using 2 Gy radiations alone.

The Expression of Bax, Bcl-2 and Caspase-3 and Fas of MCF-7 cells by Real-time PCR.

**Fig. 4:**
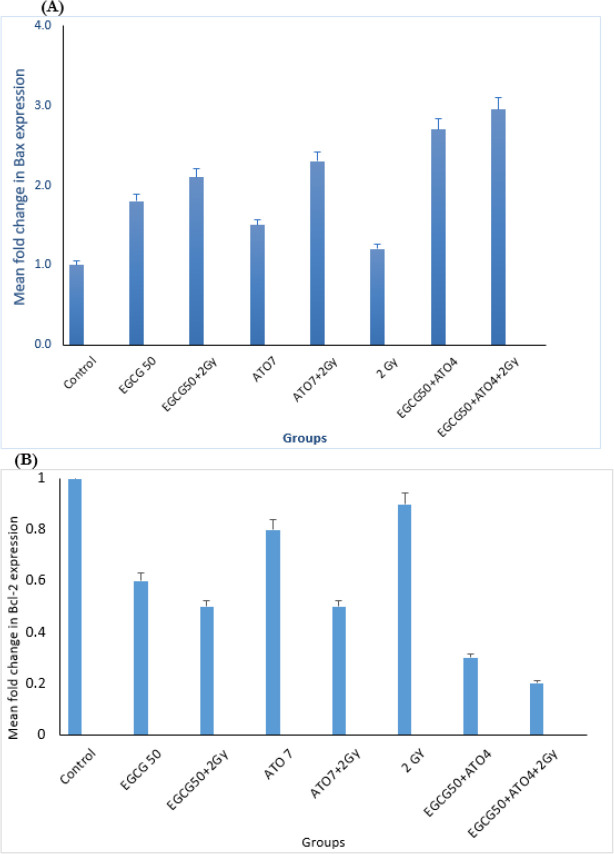
Expression status of Bax and Bcl-2. (A) Mean fold change in Bax expression. (B) Mean fold change in Bcl-2 in MCF-7 cell line following treatment by EGCG 50(μ*M*) ± IR, ATO 7(μ*M*) ± IR, EGCG50+ATO4 ±IR and 2 Gy groups

**Fig. 5:**
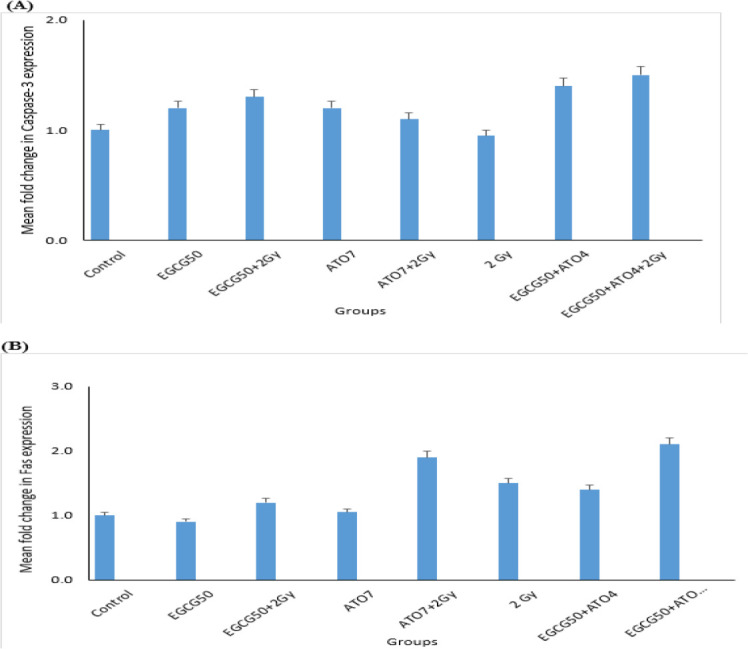
Expression status of Caspase-3 and Fas. (A) Mean fold change in Caspase-3 expression. (B) Mean fold change in Fas in MCF-7 cell line following treatment by EGCG 50(μ*M*) ± IR, ATO 7(μ*M*) ± IR, EGCG50+ATO4 ±IR and 2 Gy groups

**Fig. 6:**
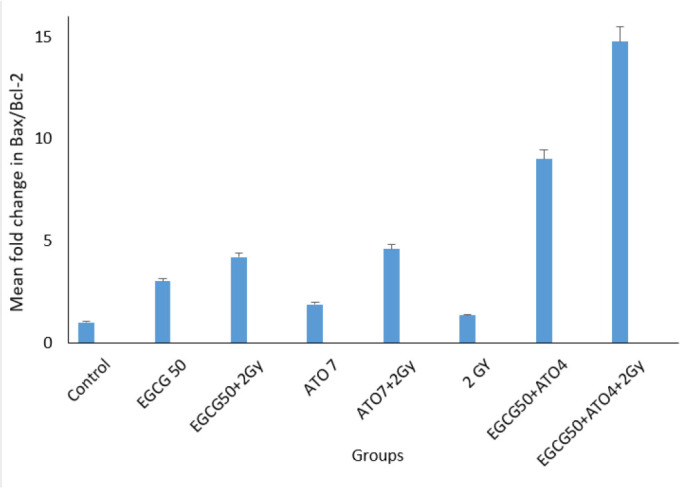
Real-time PCR analysis for the ratio of Bax/Bcl-2 among different groups

Comparison of the relative mean fold changes in gene expression levels of Bax, Bcl-2, caspase-3, Fas and Bax/Bcl-2 ratio in MCF-7 cells treated by EGCG± IR, ATO ± IR, EGCG+ATO ± IR and IR after 48 h has been shown in Figs. 4, 5 and 6. Real-time PCR showed that relative expression of Bax (Fig. 4A) was increased in all treated samples, especially in combination group (*P*≤0.05), in comparison to the control group.

The expression of the anti-apoptotic Bcl-2 gene was decreased in samples, in comparison control group (*P*≤0.05).

The Bax/Bcl-2 ratio shows the degree of vulnerability of the cells to apoptosis. Moreover, the expression level of Caspase-3 has been shown in Fig. 5 (A).

Moreover, the expression level of Fas was increased only in the presence of 2 Gy radiation groups.

## Discussion

Recent studies, have been focused on natural products mainly plants, for cancer treatment. Although, surgery, radiotherapy and chemotherapy are the most important therapies for cancer management, but the mortality rate from breast cancer remains high. In addition, the destructive and unpredictable effects of those therapeutic on normal cells are other important concerns (6). The present study, investigated the effects of EGCG and Arsenic trioxide, individually and in combination, on cell proliferation in ER-positive, MCF-7 breast cancer cells. Combination of green tea catechin with anticancer compounds such as curcumin, tamoxifen, raloxifene, resveratrol, y-tocotrienol and tricostatin enhance anticancer effects, including suppression of cell viability and colony formation, increase in catalase activity, synergistic cytotoxicity to the cells and G2/M-phase cell cycle arrest in human breast cancer cell lines (24–27).

Compound from green tea, epigallocatechin-3-gallate (EGCG) could inhibit the proliferation of over 20 different cancer lines (28). Present study approved this anti-proliferative effect of EGCG on MCF-7 cell line. The cell growth inhibition produced by green tea catechin is mediated through the induction of apoptosis (29). This study also evaluated survival rate of MCF-7 cells after treatment with Arsenic tri oxide (ATO), EGCG and radiation with MTT assay. This research showed that EGCG and ATO inhibit the proliferation of MCF-7 cells in a dose and time-dependent manner. When MCF-7 cells were treated with EGCG in a series of different doses (10 μM–100 μM) for 48 h, the survival rate of MCF-7 cells was decreased from 85% to 15%. On the other hand, single treatment with ATO in different doses (4 μM–10 μM) for 48 h, the survival rate of these cells was reduced up to 18.8%. Te-Chang Lee et al. showed dose-dependent and time-dependent effect of EGCG and ATO on HL-60 cells. Moreover, the synergistic effects of EGCG and ATO were revealed on HL-60 cells treatment. The combination of EGCG and ATO had the strong effect of suppressing invasive cells and induction of apoptosis. Our study approved these results on MCF-7 cell line (30). Therefore, the increase of cell growth inhibition by the combination of EGCG and ATO is likely to use the same mechanism for induction of apoptosis. Apoptosis is a form of programmed cell death of an organism to eliminate its unwanted cells. The mechanism of apoptosis is very complex and induction of apoptosis is widely recognized as an important tool for cancer therapy. There are 2 core pathways to induce apoptosis: extrinsic pathway that is triggered by Fas/Fas ligand composite and intrinsic pathway that is mediated by mitochondria (31). In cancer cells, apoptosis pathway is often impaired so it limits cancer therapy efficiencies considerably. Whether a cell should live or die is largely determined by anti-apoptotic regulators like Bcl-2 and Bcl-xL of Bcl-2 family and pro-apoptotic regulators like Bax and Bak. However, caspases are common between 2 pathways. This study aimed to examine if apoptosis induction plays a significant role in the EGCG dependent enhancement of ATO cytotoxicity. This study focused on some biomarkers including the Bax, Bcl-2 as the important biomarkers of the intrinsic pathway, Fas factor as extrinsic pathway and Caspase-3 as a common factor in induction of apoptosis. The results confirmed that a combination of ATO and EGCG enhances cell growth inhibition and apoptosis. We wanted to determine the minimal concentration of ATO required to be combined with EGCG for enhancement of cell death. In fact, usage of lower doses of chemotherapeutic would be desired because of their lower toxicity in the patients. Findings of this study revealed that combination of EGCG and ATO in presence of radiation also enhances the expression of Bax gene compared with the expression produced by EGCG and ATO alone. Anti-apoptotic regulators such as Bcl-2 was significantly reduced in the combined group compared with the control group. In addition, Bcl-2 expression was also significantly reduced in the combination group compared with the EGCG and ATO alone (Fig.4B). EGCG and ATO have synergistic inhibitory effects on cell proliferation of the MCF-7 cell line.

EGCG plus radiation caused the apoptotic effect and cell cycle arrest at the G_0_ /G_1_ phase in MDA-MB-231 cells. However, the results of this study have no agreement with those (32).

Germano Baj, et al, demonstrated apoptosis induction in MCF-7 and MDA-MB-231 cell lines using 0.5–5 μM ATO treatment in 24–72 hour. Our study approved that ATO in presence or in absence of IR could induce apoptosis with expression Bax and down-Regulation Bcl-2 on MCF-7 cell line (33). This study approved these results, also expression of Fas increased only in presence of radiation groups, and Caspase-3 had a little change.

The combination of EGCG and ATO would potentially be a chemopreventive factor against breast cancer. Our investigation evaluated the combined therapy of EGCG-ATO, EGCG-radiation, ATO-radiation and EGCG-ATO-radiation on MCF-7 cell line and found the synergistic effect of those methods. The expression of apoptotic genes in the combination groups was significantly higher than other groups. Experiments described in this paper strongly suggest that the anti-cancer activity of EGCG is selective and therefore may have a therapeutic value. EGCG preferentially induced apoptosis in cultured MCF-7 cell lines.

## Conclusion

The combined therapy of EGCG and ATO in presence or absence of radiation and ATO combined with radiation have synergistic effect on apoptosis induction and survival rate reduction on MCF-7 cell line. EGCG and ATO, particularly in combination, would have potential effect on the chemoprevention of breast cancer.

## Ethical considerations

Ethical issues (Including plagiarism, informed consent, misconduct, data fabrication and/or falsification, double publication and/or submission, redundancy, etc.) have been completely observed by the authors.
